# Recent HIV Infections in Italy: Data from the HIV National Surveillance System, 2012–2023

**DOI:** 10.3390/pathogens14090835

**Published:** 2025-08-22

**Authors:** Barbara Suligoi, Vincenza Regine, Lucia Pugliese, Claudio Galli

**Affiliations:** 1 National AIDS Unit, Department of Infectious Diseases, Italian National Institute of Health, 00161 Rome, Italy; barbara.suligoi@iss.it (B.S.); vincenza.regine@iss.it (V.R.); lucia.pugliese@iss.it (L.P.); 2 Independent Researcher, 00139 Rome, Italy

**Keywords:** HIV surveillance, new HIV diagnosis, recent HIV infection, RITA, CD4, HIV-RNA, late HIV diagnosis

## Abstract

The identification of recent HIV infections among newly diagnosed HIV cases is relevant to both implementing targeted prevention measures and estimating HIV incidence. We analyzed data on new HIV diagnoses in Italy from 2012 to 2023. We selected cases that were tested by at least one of three criteria (test for HIV recency, data on HIV seroconversion, clinical signs of acute HIV infection) to assess the rate of recent (<1 year) HIV infections. We analyzed these cases by gender, age group, nationality, and mode of transmission, and revaluated cases that were initially scored as a late diagnosis but then classified as a recent infection. Out of 36,289 new HIV diagnoses, 17,558 (48.8%) were tested for recent infection by at least one criterion and 3772 (21.5%) were classified as recent. At multivariate analysis, the probability of being recently infected was significantly higher among males, people aged 15–44 years, Italians, individuals diagnosed in Northern and Central Italy, heterosexual males, MSM, and people with a CD4 count ≥ 350 cells/uL at diagnosis. Of 8658 cases initially classified as late diagnoses, 979 (11.3%) were reclassified as recent by the aforementioned criteria. Monitoring recent infections among new HIV diagnoses is beneficial to individuals—because it motivates recently infected people to comply with antiretroviral treatment (which is more effective if started early) and to collaborate to partner notification, and to public health, as it provides evidence of epidemiological changes and stresses the need for targeted prevention in well-defined populations at risk.

## 1. Introduction

Since the identification of acquired immune deficiency syndrome (AIDS) and its causative agent, human immunodeficiency virus (HIV), in the 1980s [1], HIV/AIDS has represented a major global health challenge. In 2023, 39.9 million people were estimated as living with HIV globally and 630,000 HIV-related deaths have been reported [2]. In the WHO European Region, HIV infection affects the health and well-being of nearly 2.6 million people [3]. A strategy to achieve the control of HIV infection by 2030 was proposed by UNAIDS in 2023; this strategy aims to reduce new HIV infections from 165 million to less than 370,000 per year and AIDS-related deaths from 718,000 to less than 250,000 per year [4]. Progress towards the first objective may be efficiently monitored by HIV surveillance programs, which have been in place in most countries for many years. The most recent report on HIV surveillance in the WHO European Region yielded a total of 112,883 new HIV diagnoses from 47 of the 53 countries in 2023, of whom 24,731 were reported by the 30 countries of the European Union/European Economic Area (EU/EEA) [3]. Based on these data, the crude overall incidence rate of new HIV diagnoses in the European Region in 2023 was 12.7 per 100,000 population. A total of 21 out of 47 countries (44.7%) reported an increase compared to 2022, and the overall incidence of new HIV diagnoses showed a slight increase (+2.4%) compared to 2022 (12.4 per 100,000), but a 19.6% decrease compared to 2019 (15.8 per 100,000) in the pre-COVID-19 pandemic period. Accordingly, in EU/EEA countries, the incidence rate of HIV infection in 2023 was 5.3 per 100,000 inhabitants, which represents a 15.9% decrease from the 6.3 per 100,000 rate observed in 2014. However, focusing only on newly reported cases and excluding previous positive diagnoses, the rate increased by 11.8% between 2022 and 2023 (from 3.4 to 3.8 per 100,000).

The increase in new HIV diagnoses in 2023 can be attributed to various factors, such as a rebound in HIV testing and case detection after the COVID-19 pandemic, new testing policies, increased diagnoses among migrants from high-prevalence countries, and expanded HIV testing services. Among cases diagnosed in 2023 with available information on CD4 cell count, 24.0% were identified as recent infections, implying that these individuals acquired the infection within 12 months of diagnosis [5]. Discriminating recent HIV infections from established ones is relevant as the trends in the former allow us to calculate and model incidence estimates according to demographic and behavioral variables, thus helping to monitor and control the spread of the infection [6]. Moreover, identifying recent HIV infections facilitates understanding of the circumstances of HIV acquisition. People who are aware of having acquired HIV recently are motivated to reduce the risk of onward transmission. Further, they are informed of the higher efficacy of antiviral treatment when it is started shortly after infection, which leads to higher compliance with linkage to care [7].

In Italy, the surveillance system for newly diagnosed HIV infections was implemented in 2008 after a decree of the Ministry of Health [8], and it requires all regional health departments to report newly diagnosed HIV cases in a standardized format to the National AIDS Unit at the National Institute of Health (Istituto Superiore di Sanità, ISS). Complete reporting coverage for all regions was achieved in 2012.

In the present study, we considered data on recent HIV infections included in the National HIV Surveillance system from 2012 to 2023. The objective was to analyze the proportion of recent infections among new HIV diagnoses across years and, using demographic and behavioral variables, to study trends in HIV recency, epidemiological dynamics by population group, and behaviors associated with recent HIV infection. This information will be of help to health authorities at national and regional levels in shaping and tailoring interventions aimed at reducing the risk of infection and HIV incidence. Additionally, we studied the proportion of recent infections that, showing a low-level CD4 count, would be erroneously classified as a late diagnosis (i.e., HIV infections at a very late stage) in order to weigh the overestimation of late HIV diagnoses in Italian HIV surveillance data.

## 2. Materials and Methods

### 2.1. Population and Study Design

In this study, we analyzed data on newly diagnosed HIV infections from 1 January 2012 to 31 December 2023 that were reported to the National HIV Surveillance System (updated to December 2024). Data reported before 2012 were not included because the coverage of the National HIV Surveillance System was not complete prior to that year. In brief, the National HIV Surveillance System collects the following individual information:

(a) Demographic characteristics: age at diagnosis, gender, nationality, geographical area of residence.
(b) Laboratory and clinical data: date of first HIV positive test, date of last HIV negative test, first CD4 cell count, AIDS-defining conditions at the time of first HIV diagnosis, test for recent infection, clinical evidence of acute infection, clinical stage according to CDC classification.
(c) Mode of transmission: injecting drug use (IDU), heterosexual female, heterosexual male, men who have sex with men (MSM), and other/unknown.

Data on newly diagnosed HIV infections are collected in each of the 21 Italian regions or autonomous Provinces by local Health Care departments and sent annually to the National AIDS Unit at the Italian National Institute of Health (ISS). Each new case is reported anonymously using a secure platform for the online upload of data.

### 2.2. Definition of Recent Infection and Late Diagnosis

**Figure 1:**
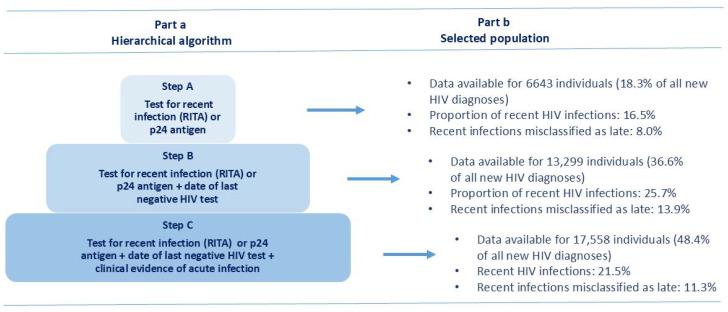
Algorithm for the selection of recent HIV infections in the study population. National HIV Surveillance System, Italy, 2012–2023. RITA = recent infection testing algorithm; p24 = HIV-1 p24 antigen.

In this study, a recent HIV infection was defined as an infection acquired less than one year before HIV diagnosis [5,9]. The identification of recent HIV infections among newly diagnosed cases was performed according to the availability of at least one of the three following criteria: (a) test for recent infection (RITA or p24 antigen); (b) date of last negative HIV test; (c) clinical evidence of acute infection (flu-like symptoms, such as fever, headache, and rash) according to the National Institute of Health [10] and other sources [11]. These criteria were applied hierarchically as three subsequent steps, and the criteria were not mutually exclusive (Figure 1, part a).

In the National HIV Surveillance System, HIV infections that are not classified as recent are scored as established, or long-standing, infections; a subset of these is defined as ‘late diagnoses’ if the CD4 cell count at first observation is <350 cells/uL or if an AIDS-defining condition is recorded regardless of the CD4 count [9]. The classification of recent HIV infection by the three criteria outlined above was applied to review the status of cases defined as late diagnoses. Every case with a CD4 count < 350 cells/uL (a late diagnosis based on CD4 count) that was classified as recent infection was considered a misclassified late diagnosis. For this purpose, we calculated the proportion of misclassification as follows: the number of people newly diagnosed with a CD4 count < 350 cells/uL or with an AIDS-defining event reclassified as recent, divided by the total number of new diagnoses with a CD4 count < 350 cells/μL or with an AIDS-defining condition.

Following the reclassification of erroneous late diagnoses, we compared the proportion of late diagnoses before and after reclassification.

### 2.3. Statistical Analysis

We analyzed the distribution of the total population by each variable (gender, age class, nationality, geographical area of diagnosis, mode of transmission, CD4 cell count at diagnosis) in the study period (2012–2023). In multivariate analysis, records with missing values in any of the variables considered were excluded from calculations.

The proportion of new diagnoses tested for recent infection and that of recent infections among those tested for recent infection were calculated, and trends over time were analyzed. Univariate analysis for recent infection was performed for all variables. Multivariate analysis was performed for variables that were significantly associated with being recent at univariate analysis, and 95% confidence intervals were calculated. A similar analysis was performed to study the probability of being misclassified as late diagnosis. The differences between proportions were evaluated using the Chi-square test (a *p* < 0.05 was considered significant). The analysis was performed using IBM SPSS Statistics 29.

## 3. Results

In the study period 2012–2023, 36,289 new HIV diagnoses were reported to the National HIV Surveillance System in Italy. The number of newly diagnosed HIV cases decreased from 2018 onwards, with a minimum in 2020, followed by an increase in the following years (Table 1). Among new HIV diagnoses, at least one criterion defining a recent HIV infection was reported for 17,558 (48.8%) cases. Data for each one of the three steps (Figure 1, part b) show that 6643 new HIV diagnoses were tested for RITA or p24 antigen (step A) of whom 16.5% were classified as recent infections; 13,299 new diagnoses had a test for RITA or p24 antigen plus the date of last HIV-negative test (step B), of whom 25.7% were classified as recent infections; finally, 17,558 new diagnoses had a test for RITA or p24 antigen plus the date of last HIV-negative test and the clinical evidence of acute infection (step C), of whom 21.5% were classified as recent infections. The overall distribution of tested cases by testing criteria (that are not mutually exclusive) shows that RITA or p24 antigen testing results were reported in 18.3% of cases, the date of last negative HIV test in 23.2%, and clinical evidence of acute HIV infection in 32.8%. Figure 1 shows the increase in tested cases following the hierarchical algorithm from step to step: from step A to B, the number of tested cases increased from 18.3% to 36.6%, and from step B to C increased from 36.6% to 48.4%.

**Figure 2:**
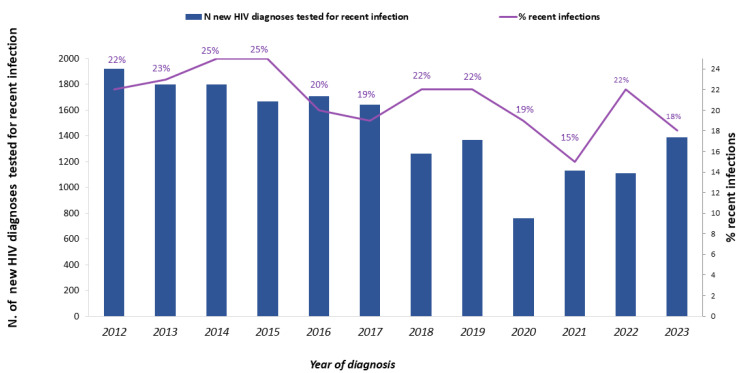
Number of new HIV diagnoses tested for recent infection (solid blue bar, left *y* axis) and percentage of recent infections (purple line, right *y* axis). National HIV Surveillance System, Italy, 2012–2023.

The number of individuals tested for recent infection with any of the described criteria decreased during the study period (Figure 2) following a trend similar to the decreasing number of total new HIV diagnoses, whereas the proportion of those tested for recent infection increased over time, from 45.8% in 2012 to 59.1% in 2023 (Table 1). In detail, between 2012 and 2023, step A of the hierarchical algorithm increased from 9.9% to 24.1%, step B remained stable over time (23.2% on average), and step C increased from 26.4% to 49.8%, respectively.

The percentage of tested individuals was similar by gender, age group, and nationality, and was significantly (*p*-value < 0.001) higher among MSM and people with CD4 count > 350 cells/uL at diagnosis (Table 2).

During the study period, 3774 new HIV diagnoses were classified as recent infections, representing 21.5% of the 17,558 tested for recent infection. The number of recent infections decreased from 429 in 2012 to 255 in 2023, and the percentage of recent infections out of those tested varied over time, with a maximum of 25% both in 2014 and 2015, and a minimum of 15% in 2021 (Table 1, Figure 2).

**Figure 3:**
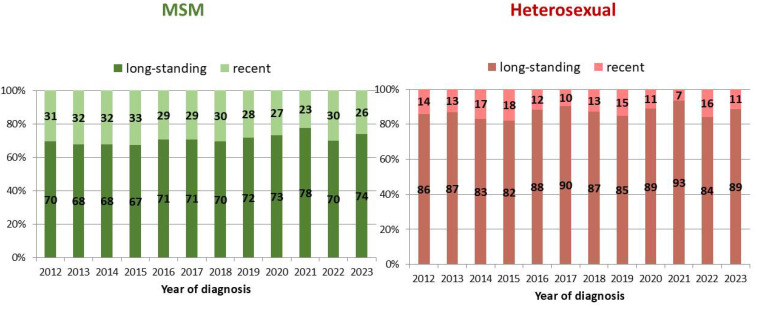
Proportion of recent and long-standing infections out of all new HIV diagnoses, by the two most frequent modes of transmission and year of diagnosis. National HIV Surveillance System, Italy, 2012–2023.

According to univariate analysis, the highest proportion of recent infections was observed among cases with CD4 count at diagnosis ≥350 cells/uL (31.2%), people aged 15–24 years (30.3%), and MSM (29.7%). Figure 3 shows the difference in the proportion of recent and long-standing infections between MSM and heterosexuals, by year of diagnosis. At multivariate analysis, the proportion of recent infections was significantly higher among males, people aged 15–44 years, Italians, individuals diagnosed in Northern and Central Italy, heterosexual males, MSM, and people with CD4 count at diagnosis ≥ 350 cells/uL (Table 2).

Recent infection data were used to reclassify new HIV diagnoses that were defined as late diagnoses based on low CD4 count < 350 cells/uL or presence of an AIDS-defining event. Out of the 17,558 that were tested for recent infection between 2012 and 2023, 8658 (49.3%) were defined as late diagnoses. Among these, 979 (11.3%) were recent infections.

**Figure 4:**
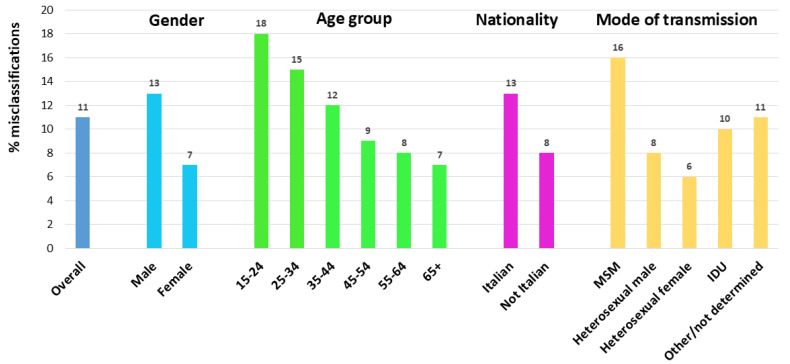
Proportion of recent HIV infections among cases misclassified as late diagnoses by gender, age group, nationality, and route of transmission. National HIV Surveillance System, Itay, 2012–2023.

**Figure 5:**
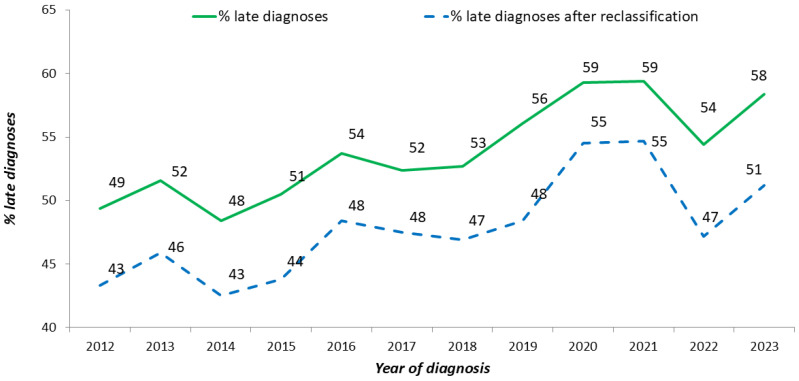
Proportion of late diagnoses (CD4 < 350 cell/uL or AIDS presenter) out of new HIV diagnoses, before (green line) and after (blue dotted line) reclassification based on recent infection testing. National HIV Surveillance System, Italy, 2012–2023.

After correcting for these misclassified cases, the overall proportion of late diagnoses decreased from 49.3% to 43.7%. The proportion of misclassified late diagnoses was significantly (*p*-value < 0.001) higher among males, Italians, MSM, and decreased with decreasing age at diagnosis (Chi-square for trend; *p*-value < 0.001) (Figure 4). Figure 5 shows the temporal trend of the percentage of late diagnoses reported to the National HIV Surveillance System, which increased from 49.4% in 2012 to 58.4% in 2023. When misclassified late diagnoses were reallocated, the proportion of late diagnoses to the total number of new HIV diagnoses decreased every year (43.3% in 2012 and 51.2% in 2023). The decrease ranged from 4% to 8% across the 12 years of the study period, and no temporal trend was observed.

## 4. Discussion

Preventive interventions to reduce the spread of HIV infection include a variety of measures, such as educational campaigns, greater access to HIV testing including opt-out strategies, early antiviral treatment, and pre- or post-exposure prophylaxis [4,12]. These measures, and a reliable evaluation of their effectiveness, require monitoring HIV incidence, which, in most Western European countries, relies on the surveillance of new HIV diagnoses. In the European WHO region, the rates of newly diagnosed HIV cases show great geographical differences, being higher in the Eastern part of the region (32.6/100,000) than in Western and Central Europe (6.2 and 4.2/100,000, respectively). New HIV infections are increasingly attributable to sexual transmission, whereas injecting drug use, though still relevant (27%), shows a decrease over time [13].

Guidelines for the measurement of HIV incidence have been issued by UNAIDS in 2011 [5] and several models have been proposed, including data derived from routine or specific HIV incidence assays [14,15,16,17,18] alone or combined with other data [5,12,19] which have proven useful to estimate incidence at a population level. The use of laboratory assays for recent HIV infections allows a quick and reliable identification of population groups that are recently infected. These target groups contribute significantly to HIV transmission because of the high HIV viral load and therefore are estimated to account for 40–60% of HIV transmission [20,21]. An accurate and fast identification of recent infections coupled with early antiviral treatment leads more rapidly to viral suppression. This will be beneficial for the individual whose life expectancy will be quite similar to that of an uninfected person [13], and for the community because HIV transmission will be substantially curbed and HIV incidence will decrease towards the ideal goal of zero new infections [17].

The analysis of Italian HIV Surveillance data carried out in this study provides some interesting information on recent HIV infections and related insights. HIV recency is generally defined as an infection that occurred a few months before the time of diagnosis [5,22,23] but the timeframe employed to define HIV recency is still a matter of debate, as it depends heavily on the criteria employed for the classification [5,12] and may vary from less than four months up to twelve months after the infection. In this study, we adopted a stepwise approach to define a newly diagnosed HIV case as ‘recent’ by following the advice of the EuroTEST HIV Late Diagnosis Definition Working Group [11] that suggests three different criteria, of which at least one must be present to identify a recent infection. The three parameters are as follows: laboratory evidence of recent infection, last HIV negative test within 12 months, and clinical evidence of acute infection.

Laboratory evidence of a recent HIV infection may include a single HIV recency test, such as the HIV avidity [13,14,16] or a combination of assays and other relevant information about the recency of HIV infection, defined as RITA (Recent Infection Testing Algorithm) [5]. To develop a RITA, it is necessary to derive the mean of the so-called ‘RITA duration’, i.e., the period interval between the time of HIV acquisition and the time that defines the infection as recently acquired by that specific algorithm. If RITAs were errorless, that period would be the same for all individuals, and every infection in a given population would be correctly classified as recent or non-recent. However, the immunological response to HIV infection differs among individuals, making RITAs not perfect; some recent infections may be classified as non-recent and vice versa. Misclassifications represent a problem especially when long-standing infections are classified as recent, since this may lead to a serious bias increasing HIV incidence estimates. For this reason, RITAs are developed to minimize the likelihood of a false recent HIV infection. For example, a RITA can start with an assay for recent infection followed by other supplementary information that may include a CD4 T cell count < 100 cells/uL, the presence of an AIDS-defining condition, a clinical record-based HIV diagnosis having occurred more than one year before and being under antiretroviral treatment (ART) [5,24]. Cases are initially evaluated by the recency laboratory assay, and those with a recent infection result are reclassified as non-recent based on the results of one or more of the other parameters.

Evidence of HIV seroconversion, based on a negative HIV testing result in the prior 12 months, is the best indicator for HIV recency, with an almost 100% accuracy. A positive HIV test result may include either a confirmed positivity for HIV specific antibodies, or positive result for HIV-RNA or the HIV p24 antigen, both being detectable before HIV antibodies in the early stages of HIV infection (Fiebig stages I and II) [25]. The only caveat on this indicator is that in the very late stages of HIV infection, the antibody response, especially towards gag-derived antigens, may be weak and the p24 antigen may become detectable due to the low levels of anti-p24 antibodies, therefore resembling a recent infection [26,27].

Clinical evidence of an acute HIV infection (AHI) satisfies, in principle, the criteria for a recent infection, but this diagnosis lacks well-defined symptoms with adequate sensitivity and specificity [28]. Symptoms are associated with inflammatory response and strong cellular activation and usually appear 1–2 weeks after the infection, last for a few weeks, and may include fever, headache, polymyalgia, pharyngitis, lymphadenitis, and skin rash, thus resembling other infections such as mononucleosis. Being AHI systemic, gastrointestinal and neurological symptoms may also appear [28,29]. These flu-like symptoms are observed in approximately 50% patients with an AHI [30], but it is difficult to estimate the frequency of asymptomatic or mild-symptomatic cases (considered as false negative cases), making the sensitivity of the clinical diagnosis low. As mentioned above, most AHI symptoms are nonspecific and can be related to other diseases (considered as false positive cases) making the specificity of AHI diagnosis based exclusively on clinical presentation debatable [29,31]. Moreover, the duration of AHI is short, and the probability for clinicians of missing AHI symptoms is relevant, therefore reducing the number of cases that would be included as recent infections.

The availability of indicators of a recent HIV infection is not uniform across Europe. A survey carried out in 2022 by the EuroTEST HIV Late Diagnosis Definition Working Group among 53 contact points for national HIV surveillance in the WHO European Region [11] shows that 94% countries collect information on AIDS-defining conditions, 89% on HIV testing history, 89% on clinical symptoms of AHI, 78% on evidence of an AHI by testing positive for HIV-RNA and/or HIV p24 antigen along with a negative HIV antibody result, but only in 38% countries newly diagnosed HIV individuals are tested for recent infection by RITA.

In Italy, information on at least one test for recent infection over the 12 years considered was available for less than half of new HIV diagnoses, probably due to an insufficient implementation of recent infection assays at the laboratory level. As a matter of fact, in Italy, no test for recent HIV infection is on the market, and laboratories that used the RITA algorithm applied modifications to fourth-generation assays based on existing literature [32] combined with additional parameters.

Of relevance, in 2020, despite the lower number of new HIV diagnoses and recent infections due to COVID-19 restrictions, the proportion of tested individuals remained high, suggesting that the reporting centers maintained a high level of commitment despite the difficult circumstances. In the post-COVID-19 years, the number of tested individuals realigned with pre-COVID-19 figures, suggesting a rapid resumption of medical care to ordinary levels in HIV services. Of interest, the reduction in the annual number of new HIV diagnoses, the proportion of individuals tested for recent infection increased over time, most probably because of the growing attention of clinicians in identifying recently infected cases and interpreting individuals’ epidemiological history [9].

We found an overall proportion of 21.5% recent infections which is lower than that reported in the UK [33], higher than that reported in Malawi [34], Botswana [35], and Kenya [36], and similar to that observed in Brazil [37] among MSM and in South Korea [38].

The number and the proportion of recently infected cases decreased after 2015 (from 25.5% in 2015 to 18.4% in 2023). The reduction in the number of recent diagnoses may indicate that fewer people got infected over time, thus denoting a decrease in HIV incidence. As an alternative, this reduction may imply several undiagnosed cases, suggesting that HIV incidence has not truly decreased. This may be the case when people who acquire HIV do not get tested promptly and remain unaware of being seropositive until they are diagnosed late. The constant increase in the percentage of late diagnoses reported in the last decade (from 53% in 2014 to 60% in 2023) by the Italian National HIV Surveillance System [9] would indirectly support the second hypothesis.

The adjusted probability of having been infected recently was twice as high among new diagnoses with CD4 count > 350 cell/uL, MSM, and people aged 15–24 years as compared, respectively, to lower CD4 levels, other transmission groups, and individuals aged >24 years.

Almost one-third of people aged 15–24 years are recently infected and show the highest probability of a recent infection compared to older age groups, similarly to what is reported in Spain, the UK, Malawi, Brazil, and Peru [33,34,37,38,39]. This may be due to a better awareness of young people of HIV sexual exposure that leads them to be tested early or more frequently; moreover, years of at-risk sexual exposure are shorter among youth because the median age for sexual debut in Italy is 17.5 years [40].

The decrease in the proportion of recent infections by age group (from 30.3% among 15–24 years to 12.0% among 65+ years) is inversely proportional to that observed among late diagnoses reported by the Italian National HIV Surveillance System (from 36.3% among 15–24 years to 79.7% among 65+ years). Among individuals aged 15–24 years, 30.3% recent infections and 36.3% late diagnoses were observed, whereas among those aged 65+, 12.0% recent infections and 79.7% late diagnoses were observed. These results suggest that the elderly are poorly sensitive to the issue of behaviors at risk for HIV. They are tested for HIV in case of comorbidities or when HIV-related symptoms are diagnosed, and therefore, the infection is in advanced stages of immune dysfunction.

MSM show the highest proportion of recent infections, as found in other studies [33,38,41,42,43], and the probability of being recently infected is twice as high as that of other key groups. This finding may be related to a higher incidence of infection in this group, or a tendency to seek HIV testing early after at-risk exposure, or a higher frequency of HIV testing.

Positivity for recent infection allows for discriminating cases diagnosed early after seroconversion that may be misdiagnosed as late diagnoses due to a low CD4 count. In fact, during the seroconversion phase, the CD4 count is similar to that of an uninfected individual; however, some people show a transient CD4 count < 350 cells/uL after seroconversion [26,44], and in the absence of other indicators, these individuals may be misclassified as late diagnoses [33]. Our results show that 11.3% of late diagnoses were recent infections, thus reducing the overall proportion of late diagnoses (LD). The annual percentage of late diagnoses showed a relatively stable level over time, suggesting that the misclassification rate is quite constant. Reclassification was more frequent among people aged 15–24 years, MSM, and Italians. Studies conducted in the UK and Belgium reported similar findings in terms of total proportion of reclassified cases and higher misclassification among young people and MSM [22,33]. Considering the high percentage of LD reported in Italy, reclassification allows us to debunk this proportion. This is relevant especially when considering the high reclassification rate calculated among individuals aged 15–24 years and MSM, which allows for remodeling the probability of late diagnosis to a lower level in these population groups. On the other hand, the low proportion of reclassified cases among the elderly and heterosexuals confirms the high quota of LD in these groups, most probably associated with a low perception of at-risk sexual behaviors.

This study is the first one in Italy that analyses the trend of recent HIV infections and the characteristics of recently infected individuals. However, a few limitations must be mentioned. First, data on recent infections were not available for all new diagnoses, therefore limiting the coverage of the information and the robustness of reported results. Second, a clinical diagnosis of AHI is hampered by the low specificity and sensitivity of clinical signs and symptoms [29,31,44], and the short duration of AHI that reduces the number of cases classified as recent infection. Third, the probability of finding recent infections is associated with ease of access to HIV testing and frequency of repeat testing; actually, the Italian new HIV diagnosis reporting form aims to investigate these aspects by collecting information on the ‘Number of HIV tests performed in the last 2 years’, but unfortunately those data are missing in most records.

## 5. Conclusions

Our results show for the first time the proportion of recent infections among new HIV diagnoses in Italy using data from the National HIV Surveillance System. Monitoring recent infections among new HIV diagnoses has provided relevant information on populations at greater risk of acquiring HIV and areas where targeted prevention should be prioritized, such as young people aged 15–24 years and MSM. From the individual perspective, knowing to have been recently infected motivates seroconverted individuals to collaborate to enhance partner notification, adhere to retention in care, and comply with ART, which is known to have greater efficacy when started as soon as possible after infection [23]. These attitudes can optimize health quality and life expectancy of people diagnosed with recent HIV infection in that they are encouraged to maximize link to care for better control of the infection. From the public health perspective, recent infection monitoring provides evidence of epidemiological changes and stresses the need for targeted prevention in well-defined populations at risk. Specifically, our results emphasize the importance of enhancing testing campaigns and awareness on sexual behaviors at risk, promoting comprehensive sexual education in schools, implementing HIV checkpoints and other community-based services, spreading updated information through social media, and developing educational programs and initiatives tailored to the demographics and transmission modes most affected by recent infections.

## Figures and Tables

**Table 1:** Number and percentage of recent infections among new HIV diagnoses and new HIV diagnoses tested for recent infection, per year. Italian National HIV Surveillance System.

Year	*n*. ^1^ of New HIV Diagnoses	*n*. ^1^ of New HIV Diagnoses with at Least One Criterion for Recent Infection	% ^2^ of New HIV Diagnoses with at Least One Criterion for Recent Infection	*n*. ^1^ of Recent Infections	% ^2^ of Recent Infections among New HIV Diagnoses With at Least One Criterion for Recent Infection
**2012**	4188	1919	45.8	429	22.4
**2013**	3859	1802	46.7	410	22.8
**2014**	3853	1802	46.8	455	25.2
**2015**	3623	1669	46.1	425	25.5
**2016**	3726	1708	45.8	340	19.9
**2017**	3619	1642	45.4	319	19.4
**2018**	3038	1261	41.5	279	22.1
**2019**	2510	1368	54.5	306	22.4
**2020**	1470	761	51.8	145	19.1
**2021**	1914	1129	59.0	172	15.2
**2022**	2140	1108	51.8	239	21.6
**2023**	2349	1389	59.1	255	18.4
**Total**	**36,289**	**17,558**	**48.4**	**3774**	**21.5**

^1^ *n*. = number; ^2^ % = percentage.

**Table 2:** Number of new HIV diagnoses, newly diagnosed cases tested for recent infection, proportion of recent infections and logistic model (crude and adjusted) for being recently infected. Data from the Italian National HIV Surveillance System, 2012–2023.

	*n*. ^1^ of New HIV Diagnoses	*n*. ^1^ of New HIV Diagnoses with at Least 1 Test for Recent Infection	% ^2^ of New HIV Diagnoses with at Least 1 Test for Recent Infection	*n*. ^1^ of Recent Infections	% ^2^ of Recent Infections out of New HIV Diagnoses with at Least 1 Test for Recent Infection	OR ^3^ (95% C.I.)	aOR ^4^ (95% C.I.)
**Total**	**36,289**	**17,558**	**48.4**	**3774**	**21.5**		
**Gender**							
Female	7950	3582	45.1	430	12.0	1	
Male	28,339	13,976	49.3	3344	23.9	2.3 (2.1–2.6) *	
**Age**							
15–24	3096	1411	45.6	427	30.3	3.2 (2.4–4.2)	2.2 (1.7–3.0) *
25–34	10,242	5215	50.9	1377	26.4	2.6 (2.0–3.4)	1.8 (1.4–2.4) *
35–44	10,257	5083	49.6	1101	21.7	2.0 (1.6–2.6)	1.5 (1.2–2.0) §
45–54	7692	3684	47.9	593	16.1	1.4 (1.1–1.8)	1.1 (0.9–1.5)
55–64	3487	1550	44.5	197	12.7	1.1 (0.8–1.4)	1.0 (0.7–1.3)
65+	1353	569	42.1	68	12.0	1	1
unknown	162	46	28.4	11	23.9		
**Nationality**							
Italian	25,092	12,393	49.4	3022	24.4	1.9 (1.7–2.1)	1.7 (1.5–1.9) *
Non-Italian	11,028	5109	46.3	741	14.5	1	1
unknown	169	56	33.1	11	19.6		
**Geographical area of diagnosis**							
North	18,324	9728	53.1	2379	24.5	1.8 (1.6–2.0)	1.7 (1.5–1.8) *
Center	9892	3964	40.1	804	20.3	1.4 (1.3–1.6)	1.3 (1.1–1.4) *
South and Islands	8073	3866	47.9	591	15.3	1	1
**Transmission mode**							
Heterosexual females	6736	3177	47.2	367	11.6	1	1
Heterosexual males	9339	4005	42.9	585	14.6	1.3 (1.1–1.5)	1.4 (1.2–1.6) *
IDU ^5^	1411	767	54.4	118	15.4	1.4 (1.1–1.7)	1.2 (0.9–1.5)
MSM ^6^	14,481	8461	58.4	2517	29.7	3.1 (2.9–3.6)	2.3 (2.0–2.6) *
Other/unknown	4322	1148	26.6	187	16.3	1.5 (1.2–1.8)	1.4 (1.2–1.8) *
**CD4 cell count at diagnosis**							
<350 cell/mL	17,128	8509	49.7	958	11.3	1	1
≥350 cell/mL	13,219	7716	58.4	2404	31.2	3.5 (3.1–4.0)	2.8 (2.6–3.1) *
unknown	5942	1333	22.4	412	30.9		

^1^ *n*. = number; ^2^ % = percentage; ^3^ OR = odds ratio; ^4^ aOR = adjusted odds ratio for multiple logistic model including the following variables: age, nationality, geographical area of diagnosis, transmission mode, CD4 cell count; ^5^ IDU = injecting drug use; ^6^ MSM = men having sex with men; * = *p*-value < 0.001; § = *p*-value < 0.005.

## Data Availability

No new data were created or analyzed in this study, and the database utilized is restricted to authorized users and cannot be shared.

## Abbreviations

The following abbreviations are used in this manuscript:

AIDS	Acquired Immune Deficiency Syndrome
HIV	Human Immunodeficiency Virus
WHO	World Health Organization
UNAIDS	Joint United Nations Programme on HIV/AIDS
EU/EEA	European Union/European Economic Area
COVID-19	Coronavirus 19 (SARS-CoV-2)
ISS	[Istituto Superiore di Sanità] National Institute of Health
IDU	Injecting Drug Users
MSM	Men who have sex with men
RITA	Recent Infection Testing Algorithm
ART	Antiretroviral treatment
LD	Late HIV diagnoses
